# Laser-based therapies in the management of non-melanoma skin cancers: a narrative review

**DOI:** 10.1007/s10103-026-04899-0

**Published:** 2026-06-06

**Authors:** Francesco Russano, Davide Brugnolo, Luigi Dall’Olmo, Francesco Callegarin, Paolo Del Fiore, Marcodomenico Mazza, Alberto Camuccio, Daniele Scafuri, Davide Furlan, Marco Rastrelli, Simone Mocellin

**Affiliations:** 1 Soft-Tissue, Peritoneum and Melanoma Surgical Oncology, Veneto Institute of Oncology IOV-IRCCS, Padua, Italy; 2 Department of Surgery, Oncology and Gastroenterology (DISCOG), University of Padua, Padua, Italy; 3 Clinical Research Unit, Veneto Institute of Oncology IOV-IRCCS, Padua, Italy; 4 Immunology and Molecular Oncology Diagnostics Unit, Veneto Institute of Oncology IOV-IRCCS, Padua, Italy

**Keywords:** Non-melanona skin cancers, Laser, Basal cell carcinoma, Cutaneous squamous cell carcinoma

## Abstract

The rising global incidence of basal cell carcinoma (BCC) and cutaneous squamous cell carcinoma (cSCC) necessitates the exploration of tissue-sparing alternatives to surgical excision, which remains the gold standard. Laser systems (CO₂, Nd: YAG, PDL) offer a less invasive approach using selective photothermolysis, addressing functional and aesthetic concerns in anatomically sensitive areas. This narrative review summarizes the current clinical data on laser-based strategies for non-melanoma skin cancers (NMSC), focusing on treatment parameters, patient/lesion selection, oncologic outcomes (clearance and recurrence), and safety. A targeted search of PubMed, Embase, and Scopus up to May 2026 was synthesized narratively. For low-risk BCC, Nd: YAG lasers showed a 3.1% recurrence rate (7.9-year follow-up), while CO₂ lasers reported 9.4% (2.1-year follow-up). Pulsed dye laser (PDL) monotherapy was less reliable, with 38% recurrence. Evidence for cSCC remains limited, focusing on in situ disease and combined regimens. Integrating Reflectance Confocal Microscopy (RCM) and Laser-Assisted Drug Delivery (LADD) significantly improves oncologic efficacy and treatment depth. Laser therapy, particularly Nd: YAG and CO₂ systems, represents a viable tissue-sparing option primarily for low-risk BCC. However, evidence for cSCC remains limited and suggests higher recurrence rates, necessitating cautious patient selection.

## Introduction

Non-melanoma skin cancers (NMSCs), predominantly basal cell carcinoma (BCC) and cutaneous squamous cell carcinoma (cSCC), rank among the most frequently diagnosed malignancies worldwide [1]. Their incidence is steadily increasing in fair-skinned populations, particularly in regions with high cumulative ultraviolet exposure, aging demographics, and a growing prevalence of immunosuppression related to organ transplantation or immunomodulatory therapies [2]. While NMSCs are rarely life-threatening, their tendency to recur and the potential for substantial aesthetic and functional impairment, especially in anatomically delicate areas such as the face, underscore the need for precise tissue-preserving treatment strategies.

Surgical excision remains the gold standard for managing both BCC and cSCC, particularly in high-risk lesions, as it provides histological margin assessment and is associated with low recurrence rates. Nonetheless, surgery may not always be feasible or preferred, whether due to patient comorbidities, advanced tumor location (anatomically critical or difficult areas (e.g. the periorbital region, the nasolabial fold), or concerns about cosmetic and functional outcomes. In anatomically sensitive areas such as the periorbital, perinasal, or auricular regions, surgical interventions can lead to considerable morbidity or noticeable aesthetic alterations. Moreover, in elderly or medically fragile patients, less invasive alternative therapies may be more appropriate with several studies on octogenarian and nonagenarians demonstrating safety. However, less invasive techniques are preferred, regardless of age. In this setting, laser-based therapies like CO₂, erbium: YAG, and pulsed dye lasers (PDL) have attracted growing interest as both definitive and adjunctive treatment modalities for selected cases of NMSC [3].

Ablative systems, such as CO2 and Er: YAG, target tissue water to achieve precise vaporization. Conversely, PDL targets the tumor’s specialized microvasculature, providing a less invasive treatment option. While these technologies offer superior aesthetic outcomes and reduced morbidity, especially in anatomically sensitive regions like the eyelids, their primary clinical limitation remains the lack of histopathological margin analysis [4]. Because this absence of margin control can lead to variable recurrence rates in deeper or more aggressive malignancies, laser therapy is currently most indicated for superficial lesions, field cancerization, or patients who are poor candidates for invasive surgery [4–7]. Among these, ablative CO₂ lasers are widely utilized in managing low-risk BCCs due to their versatility; however, clinicians must consider reported recurrence rates and prioritize aggressive multi-pass protocols to optimize outcomes [8, 9].Recent data, including a systematic review of over 4,750 BCC cases, reported recurrence rates as low as 3.1% for Nd: YAG and 9.4% for CO₂ laser ablation, highlighting their potential in selected scenarios [10].

However, evidence supporting laser therapy for cSCC remains far more limited and is generally restricted to in situ disease. Small prospective studies and pilot clinical trials have reported substantial tumor regression and histologic necrosis, particularly when microneedling or fractional ablation was used to facilitate drug delivery [11–13].

Technological advancements, such as the integration of reflectance confocal microscopy (RCM) for “optical biopsies” and laser-assisted drug delivery (LADD), are currently addressing the diagnostic limitations of non-excisional methods. By modulating the tumor microenvironment and improving drug penetration, these strategies aim to enhance oncologic efficacy [14–16]. These innovations enable more accurate targeting, minimize collateral tissue damage, and may offer synergistic potential with immunomodulatory therapies, although their application remains largely investigational.

Given the expanding body of evidence and the need for clinically oriented recommendations, this narrative review summarizes current data on laser-based therapeutic strategies in cutaneous oncology, focusing on treatment parameters, patient/lesion selection, oncologic outcomes, safety, and cosmetic results.

## Methods

### Review approach

This article is a narrative review that synthesizes the available evidence on laser-based therapies for BCC and cutaneous squamous cell carcinoma cSCC. The review emphasizes clinically actionable elements, including laser parameters, adjunctive technologies, patient/lesion selection, oncologic outcomes (clearance and recurrence), safety, and cosmetic results.

### Evidence sources and search approach

We conducted a targeted, non-systematic search of PubMed/MEDLINE, Embase, and Scopus from inception to May 2026 using combinations of terms related to non-melanoma skin cancer and laser modalities (e.g., CO₂, Nd: YAG, pulsed dye laser, Er: YAG, fractional laser, laser-assisted drug delivery, and photodynamic therapy). Additional records were identified by manual screening of reference lists of key reviews and clinically influential studies.

### Selection and narrative synthesis

Because the objective was an expert narrative synthesis rather than a comprehensive systematic review, publications were selected based on relevance to clinical practice, methodological quality, and the extent to which they reported treatment parameters and follow-up. Systematic reviews, randomized or prospective comparative studies, and larger observational series were prioritized. Preclinical or technological studies are discussed separately as future perspectives. No de novo quantitative pooling or formal risk-of-bias assessment was performed; when pooled recurrence estimates are discussed, they are explicitly attributed to the original systematic reviews.

### Clinical evidence

The available clinical evidence on definitive laser-based treatment for NMSC is heterogeneous, consisting predominantly of observational cohorts and case series, with a limited number of randomized or prospective comparative studies. Most data address low-risk BCC; evidence for cSCC is more limited and mainly focused on cSCC in situ (Bowen’s disease) and on combined or adjunctive approaches, such as PDT and laser-assisted drug delivery (LADD). The current landscape of laser efficacy in NMSC is largely defined by the meta-analysis by Sharon et al. (2021) [10], which synthesized data from over 4,750 cases. Their findings, summarized in Table 2, provide the baseline recurrence rates against which newer, more specialized protocols are compared.

## Basal Cell Carcinoma (BCC)

For low-risk BCC, ablative CO₂ laser monotherapy is the most frequently reported approach. While pooled data indicates a 9.4% recurrence rate for CO₂ (Table 2), individual series demonstrate that outcomes are highly sensitive to the number of passes and margin strategies [10]. Research indicates that laser efficacy is highly dependent on histological subtype; while superficial BCCs can be reliably ablated, provided the middle dermis is reached, nodular and aggressive infiltrative subtypes are associated with significantly higher recurrence rates. The precision of the margin strategy also remains a critical factor in long-term success. For example, treating a 4-mm margin with multiple passes of a pulsed CO₂ laser has demonstrated a cure rate of 97%, comparable to traditional surgical excision [17]. Conversely, a single-pass technique without margin overlap resulted in a recurrence rate of 50%, underscoring the necessity of more aggressive, multi-pass protocols to achieve complete eradication [18]. Furthermore, recent advancements have integrated intraoperative pathologic assessment with superpulsed CO₂ laser therapy, a method particularly effective for high-risk periorbital lesions. This approach allows for immediate retreatment until no malignant cells remain, achieving a 95.2% cure rate while preserving essential lid function. Finally, the duration of follow-up is essential for an accurate oncologic assessment, as clinical recurrences in laser-treated lesions have been documented as late as 32 to 36 months post-intervention. Consistent long-term clinical evaluation is therefore vital to distinguishing between true tumor clearance and late-stage recurrence or new primary growth in the treated area [19, 20]. The oncologic efficacy of laser therapy is highly dependent on lesion selection, with single-digit recurrence rates reported in clinical cohorts limited to well-circumscribed, superficial, or nodular BCCs. For instance, studies employing aggressive multi-pass protocols have achieved cure rates reaching 97% for these specific subtypes. Conversely, significantly higher recurrence is observed in less selected groups or when treating aggressive histotypes, such as infiltrative or micronodular BCCs, which are generally deemed unsuitable for laser monotherapy due to the risk of incomplete eradication. Furthermore, the treatment of SCC in situ is frequently complicated by its greater average depth and thicker stratum corneum, which can impede complete neoplastic vaporization and lead to persistent disease [18, 21–23].

Campolmi et al. [9] reported a series of 140 patients treated with a super-pulsed CO₂ laser with no recurrences after 1.7 years of follow-up. The encouragingly low recurrence rates observed here must be contextualized by the specific case selection and protocols employed; furthermore, their long-term generalizability remains contingent on follow-up duration.

While specific cohorts demonstrate high efficacy, less favorable outcomes have been documented, highlighting the extreme sensitivity of CO₂ laser results to both protocol heterogeneity and lesion characteristics. For instance, a prospective study of 51 lesions reported a 33% failure rate, concluding that while superficial subtypes were reliably ablated, larger nodular tumors exceeding 10 mm often resulted in incomplete eradication unless the lower dermis was reached [22]. Similarly, high-sensitivity histological analysis of 17 specimens revealed that approximately 29% of cases retained residual tumor at the deep margins; notably, these failures were strictly associated with protocols utilizing only two laser passes, whereas a three-pass strategy achieved 100% clearance for superficial subtypes. Furthermore, historical data from a cohort of 25 lesions cited recurrence rates as high as 50%, a significant failure primarily attributed to an overly conservative technique involving a single, non-overlapping pass. Collectively, these findings underscore that successful tumor control is highly contingent upon employing an aggressive multi-pass delivery mode and restricting treatment to well-circumscribed superficial malignancies [18, 22, 23].

In a comparative clinical study involving 100 patients with facial BCC, the therapeutic efficacy of CO₂ laser treatment was evaluated against conventional surgical excision. The overall efficacy rate was 94% in the CO₂ laser group and 90% in the surgical group, with no statistically significant difference between the two modalities. The recurrence rate was significantly lower in the CO₂ laser group (4%) compared with the surgical group (16%). Furthermore, postoperative pain scores were markedly reduced in patients treated with the CO₂ laser, while those who underwent surgical excision reported significantly higher levels of satisfaction [24]. Pilot and proof-of-principle studies have established the feasibility of RCM as a high-resolution tool to guide CO₂ laser ablation, effectively addressing the limitations of conventional visual and dermoscopic guidance [13]. Dermoscopy remains prone to false-negative rates of approximately 20% due to non-specific post-treatment oncological features. RCM provides real-time, non-invasive identification of specific neoplastic criteria—such as basaloid tumor islands—with a resolution nearly comparable to that of histopathology. This precision enables the detection of residual disease that is otherwise clinically occult, facilitating targeted laser retreatment and ensuring oncologic radicality. Clinical evidence, including cases of nodular BCC treated with combined superpulsed CO₂ and PDT, has demonstrated successful tumor-free survival at follow-ups up to 30 months when monitored by RCM [25]. However, this technique has two major limitations: a maximum penetration depth of 200 μm and the high degree of specialized training required for accurate image interpretation. Despite the potential for significantly reduced recurrence rates compared to conventional guidance, existing literature is primarily confined to individual case studies and exploratory series. Consequently, robust comparative trials with long-term follow-up are essential to validate RCM-guided laser therapy as a standard non-surgical alternative [25].

Long-pulsed Nd: YAG laser therapy has been proposed as a definitive option for selected low-risk BCC. Nd: YAG lasers have shown the lowest pooled recurrence rates among laser modalities (3.1%, Table 2) [10]. Additional clinical studies have reported high early clearance, including a complete response rate of 92% at 1 month for lesions smaller than 1.5 cm in one series [26].

Clinical evidence from multiple cohorts and case series indicates that the 1064 nm Nd: YAG laser is a highly effective non-surgical option for low-risk BCCs, with reported histologic clearance rates consistently exceeding 90% and reaching as high as 97.3% in long-term follow-up studies [27, 28]. A notable hallmark of this modality is its high level of patient tolerability; multiple investigations have successfully performed the procedure without local anesthesia, as the associated burning sensation is generally manageable. Aesthetic results are also frequently reported as superior to traditional surgery, often yielding soft, minimally visible scars or an essentially scarless appearance, particularly in non-facial locations. However, the literature reveals a significant lack of uniformity regarding technical parameters and the underlying mechanisms of tumor destruction. Further heterogeneity exists in coagulation-based approaches that employ high-power pulses (up to 40 W) for 1–3 s to achieve a depth of necrosis up to 5 mm. Therapeutic outcome definitions also vary substantially across clinical series, ranging from simple clinical disappearance and photography-based follow-up to rigorous histopathological confirmation via post-treatment excision or serial-sectioned biopsies. This absence of a standardized protocol—encompassing pulse duration, energy density, and the number of treatment sessions—remains a barrier to the routine implementation of Nd: YAG systems as a first-line alternative to the surgical gold standard [29–31].

The integration of nonablative 1,064-nm Nd: YAG laser therapy with optical coherence tomography (OCT) represents a significant advancement in the nonsurgical management of BCC, providing enhanced precision through real-time margin assessment and continuous subclinical monitoring. In a retrospective analysis of 119 lesions, this combined approach achieved a 100% clearance rate two months after the final treatment session, with approximately 70.4% of cases requiring only a single intervention. While clinical recurrence at one year was limited to 1.7%, the utilization of OCT facilitated the identification of a cumulative 4.2% subclinical recurrence rate, detecting residual tumor islands up to 2 mm beneath the surface that were not visible to the naked eye [32]. Factors significantly associated with a suboptimal response included immunosuppression, focal sclerosing subtypes, and lesions exceeding 5 mm in diameter. By transitioning from aggressive, broadly destructive parameters to milder, imaging-guided protocols, this modality minimizes recovery time and optimizes aesthetic outcomes. Furthermore, the synergy between non-invasive imaging and laser platforms supports a highly efficient clinical workflow, enabling an integrated, single-session diagnostic and therapeutic workflow model of same-day diagnosis and treatment, which is particularly beneficial for patients in underserved areas [32].

However, prospective data on the efficacy and aesthetic outcomes of nonablative laser therapy are not entirely concordant. A recent prospective, single-center trial evaluating 1064 nm long-pulsed Nd: YAG laser treatment for BCC with a 12-month follow-up revealed significantly less favorable results than previously reported [33]. In an unselected cohort of 78 lesions, the rate of incomplete tumor clearance (ITC) was as high as 30.8% at the 3-month mark, eventually settling at a cumulative failure rate of 35.9% for those requiring multiple interventions [33]. These findings suggest that the clinical efficacy of Nd: YAG may be overestimated in studies with shorter follow-up periods or less sensitive monitoring techniques. The study also highlighted a more guarded cosmetic profile, with 46.7% of treated lesions exhibiting moderate to severe scarring after one year. Notably, patient satisfaction (77.8%) did not necessarily correlate with these objective aesthetic outcomes, as many individuals prioritized the time-saving same-day treatment model approach of combining diagnosis and treatment in a single session. These discrepant results, despite the use of identical laser parameters to earlier successful series, underscore the critical importance of protocol standardization and meticulous lesion selection. Factors such as tumor thickness (limited to < 1.2 mm in this series) and the use of OCT for entire-lesion surveillance are essential to identifying residual disease in marginal areas, which accounted for the majority of incomplete clearances [33].

Combination regimens have been explored to improve both clearance and cosmetic outcome. In the treatment of BCC, combined ER: YAG laser plus PDT has been reported to achieve high efficacy (reported overall efficacy 98.97%), with the combined approach also providing the best aesthetic results in the cited comparative experiences [34, 35].

In contrast, PDL monotherapy has demonstrated variable and generally less reliable oncologic outcomes. PDL remains the least reliable monotherapy, with meta-analysis data showing a 38% recurrence rate (Table 2). Its use is currently restricted to highly selected superficial lesions [10]. Randomized controlled trials have demonstrated that PDL can achieve complete clinical and histological remission in 78.6% of superficial BCCs located at low-risk sites. However, effectiveness declines sharply with increased tumor size; while lesions smaller than 1.5 cm show clearance rates up to 91.7%, larger tumors often exhibit incomplete responses, with clearance falling to approximately 25% [36]. Protocol modifications play a decisive role in enhancing these outcomes. Research indicates that double-stacked pulses and larger spot sizes (10 mm) significantly improve tumor destruction compared to single-pass settings by creating greater thermal damage within the tumor’s supporting microvasculature [37]. Despite the promise of an essentially scarless result and high patient satisfaction, the integration of sensitive monitoring tools, such as OCT, has revealed a substantial rate of subclinical incomplete clearance, reaching 30.8% in some series, that conventional visual exams may fail to detect [33]. Ultimately, while PDL is a viable nonsurgical alternative, its success remains contingent on rigorous lesion selection and optimized treatment parameters [38–40]. Accordingly, PDL is generally not favored as a stand-alone definitive treatment for BCC.

Laser-assisted drug delivery (LADD), typically using ablative fractional laser (AFL) to enhance penetration of topical agents (e.g., 5-fluorouracil or imiquimod), has been evaluated in small cohorts for superficial BCC, often reporting high short-term response rates. However, follow-up is frequently limited (commonly 9–12 months), and long-term recurrence data remain sparse [41].

Although not a laser-ablative modality per se, light-based photodynamic therapy is frequently discussed alongside laser approaches in tissue-sparing management of superficial NMSC. A multicenter, double-blind, phase III randomized trial published in 2025 evaluated red-light PDT with a 10% 5-aminolevulinic acid (ALA) gel for BCC and reported histological clearance of 75.9%, with favorable cosmetic outcomes [42].

Additional, less common technologies have been explored in selected anatomic sites. The dual-wavelength copper vapor laser (CVL) has been reported as effective for periorbital BCC in a limited clinical experience, with no recurrences observed over a two-year follow-up in the cited report [43].

Preclinical and translational work suggests future perspectives, such as gold nanoparticles combined with near-infrared (NIR) laser exposure (photothermal therapy) [44]. Such approaches have shown tumor reduction in murine models bearing human BCC xenografts, but remain investigational and cannot be extrapolated to definitive clinical management at present [44].

## Cutaneous Squamous Cell Carcinoma (cSCC)

Evidence for cSCC is more limited than for BCC and is largely focused on cSCC in situ (Bowen’s disease) and on combined or adjunctive approaches. Evidence for cSCC is notably weaker, with pooled recurrence estimates reaching 22% for CO₂ ablation in squamous cell carcinoma (Table 2) [10]. Beyond these pooled estimates, individual cohort studies have evaluated ablative CO₂ protocols for Bowen’s disease, including combined regimens such as ablative CO₂ followed by methyl aminolevulinate (MAL) PDT [45].

Additional reviews and case-based reports suggest that CO₂ ablation may be most effective for superficial intraepidermal disease and for selected lesions without marked follicular extension, hyperkeratosis, or significant adnexal involvement, features that may reduce response and increase the risk of persistence or recurrence. Data on definitive treatment of invasive cSCC with laser monotherapy are scarce. Most published evidence addresses adjunctive or field-directed strategies (e.g., LADD for actinic damage) rather than definitive oncologic management. A 2023 study reported a reduction in photodamage with repeated treatments, but these outcomes should not be conflated with tumor clearance endpoints [46]. Furthermore, for invasive cSCC, definitive laser monotherapy lacks sufficient evidence and should be avoided outside of clinical trials or advanced multidisciplinary settings.

### Safety and cosmetic outcomes

Across published clinical series, adverse events after laser-based treatment are generally mild and transient, most commonly including erythema, edema, oozing/crusting, pain, and temporary dyspigmentation; scarring is reported less frequently but depends on lesion depth, energy settings, and wound care. When reported, patient satisfaction and cosmetic outcomes are often favorable in appropriately selected lesions, particularly in cosmetically sensitive areas, although outcome measures are heterogeneous and not consistently validated (Table 1).

**Table 1:** Summary of clinical studies and evidence synthesis for laser-based treatment of NMSC

Reference	Study Type	Clinical Focus	Key Findings/Level of Evidence	Adverse Events/complications and tolerability
Schlesinger et al. [42]	Phase III Randomized Controlled Trial (RCT)	Superficial BCC	75.9% histological clearance using 10% ALA gel with red-light PDT.	Mild erythema and pain.
Chen et al. [24]	Comparative Clinical Study	Facial BCC	CO₂ laser showed higher efficacy (94%) and lower recurrence (4%) compared to surgery.	Markedly reduced postoperative pain compared to surgical excision.
Kranz et al. [33]	Prospective Clinical Trial	BCC (Nd: YAG + OCT)	35.9% cumulative failure rate; highlighted the need for sensitive imaging to detect residual disease.	Moderate to severe scarring reported in 46.7% of treated lesions.
Markowitz & Bressler [32]	Retrospective Analysis	BCC (Nd: YAG + OCT)	100% clearance at 2 months; 1.7% recurrence at 1 year.	Minimized recovery time and optimized aesthetic outcomes.
Karsai et al. [39]	Randomized Controlled Trial (RCT)	Superficial BCC	78.6% complete remission using PDL.	Essentially scarless results and high patient satisfaction.
Campolmi et al. [9]	Case Series	BCC	0% recurrence over 1.7 years using super-pulsed CO₂ laser.	Low rates of scarring and dyspigmentation.
Ebrahimi et al. [20]	Clinical Series	Periorbital BCC	95.2% cure rate utilizing intraoperative pathologic assessment with CO₂ laser.	Preserved essential eyelid function.
Smucler & Vlk [34]	Comparative Clinical Study	Nodular BCC	Er: YAG plus PDT achieved 98.97% overall efficacy.	Best aesthetic results among compared modalities.
Hibler et al. [13]	Pilot Proof-of-Principle Study	BCC (RCM Guided)	RCM allowed real-time detection of residual neoplastic features during CO₂ ablation.	Minimized collateral tissue damage due to high precision.
Wenande et al. [41]	Explorative Imaging-Guided Trial	BCC	Evaluation of laser-assisted drug delivery (LADD) for cisplatin and 5-FU.	Accelerated clinical healing and reduced risk of permanent scarring.
Klyuchareva et al. [43]	Case Series	Periorbital BCC	Copper vapor laser (CVL) was effective with no recurrences over 2 years.	Generally mild and transient erythema or edema.

Evidence from systematic reviews of PDT indicates that, for selected superficial lesions, PDT can achieve meaningful clearance with favorable cosmetic outcomes, although recurrence rates may be higher than with some topical agents or surgery depending on lesion subtype and follow-up duration [47]. In the context of laser-based management, PDT is most relevant as an adjunct or combination partner (e.g., post-ablative CO₂) rather than as a direct comparator to ablative laser protocols.

We summarized the main results of clinical and non-clinical studies in Tables 1 and 2.

**Table 2:** Supporting context: epidemiology, clinical guidelines, and preclinical innovations

Reference	Research Type	Content Area	Notable Insights
Sharon et al. [10]	Systematic Review & Meta-analysis	BCC and cSCC	Reported pooled recurrence rates: 3.1% (Nd: YAG), 9.4% (CO₂), and 38% (PDL).
Pesnel et al. [44]	Preclinical (Murine Model)	Photothermal Therapy	Demonstrated tumor reduction in human BCC xenografts using gold nanoparticles and NIR laser.
Wu et al. [1]	Epidemiological Report	Burden of BCC	Analyzed the increasing incidence and public health burden of BCC in the USA.
Morton et al. [50]	Clinical Guidelines	PDT Protocols	European Dermatology Forum guidelines for topical PDT in Bowen’s disease and BCC.
Zhang et al. [48]	Review	Nanotechnology & PDT	Overview of advancements in using nanosystems to enhance drug penetration in skin cancer.
Ahluwalia et al. [7]	Narrative Review	Laser Therapeutics	Discussed the evolution of laser platforms for BCC management.
Soleymani et al. [4]	Narrative Review	NMSC Analysis	Critical analysis of laser therapy indications for non-melanoma skin cancer.
Diffey & Langtry [2]	Demographic Report	Aging and Skin Cancer	Correlated skin cancer incidence with aging fair-skinned populations.
Ma et al. [14]	Review	Immunotherapy	Discussed multifunctional nanosystems for photodynamic immunotherapy.

## Adjunctive technologies

Recent evidence suggests that the integration of Reflectance Confocal Microscopy (RCM) significantly enhances the precision of CO₂ laser ablation by providing real-time, non-invasive guidance. Traditionally, the adoption of laser-based interventions has been limited by the inability to histologically confirm tumor clearance. RCM addresses this challenge by allowing clinicians to map tumor margins at cellular-level resolution both pre-ablation and in between laser passes. In a pilot study, this approach enabled the detection of residual neoplastic features, such as palisading nuclei and tumor clefting, that are typically invisible to the naked eye. By facilitating the targeted removal of these persistent nests, RCM-guided ablation has achieved complete clearance in a majority of treated cases, with results subsequently verified by definitive surgical histology. Consequently, this synergistic strategy offers a promising alternative for managing low-risk basal cell carcinomas, particularly in patients who prioritize superior cosmetic outcomes or are unsuitable for traditional invasive surgery [13]. Ablative fractional laser-assisted drug delivery (AFL-LADD) utilizes far-infrared laser systems, specifically CO₂, Er: YAG, or Er: YSSG, to create microscopic vertical pores in the epidermis. This technique effectively bypasses the stratum corneum, which typically acts as a barrier to the penetration and bioavailability of hydrophilic photosensitizers like 5-ALA or MAL [48]. Clinical trials have demonstrated that these intensified protocols are significantly more effective than standard PDT across various malignancy grades. For instance, combining fractional CO₂ lasers with advanced nanoemulsion formulations has yielded complete response rates of 93.4% in non-aggressive BCCs [49]. Furthermore, specific nonablative platforms integrated with imaging have reported clearance rates reaching 100% for selected cutaneous malignancies. By vaporizing target tissue in a matrix mode, AFL-LADD preserves areas of healthy skin between the treated zones, promoting accelerated clinical healing and reducing the risk of permanent scarring compared to traditional broad-surface ablation [48]. The integration of carbon dioxide CO₂ laser ablation with PDT has emerged as a high-efficacy, low-cycle treatment strategy for nonmelanoma skin cancer, particularly for nodular basal cell carcinomas (nBCC). While conventional PDT is often limited by a maximum light penetration depth of 2 mm, using a superpulsed CO₂ laser to debulk the lesion allows clinicians to reach the reticular dermis and effectively bypass the epidermal barrier. This pretreatment significantly enhances the absorption and accumulation of photosensitizers like 5-ALA or MAL into the deeper neoplastic layers. Clinical studies have demonstrated that this combined regimen can achieve recurrence-free clearance rates ranging from 97% to 100% [50]. A primary advantage of this approach is the reduction in the total number of required treatment sessions; whereas traditional protocols for thick or recalcitrant lesions may require multiple cycles, the laser-PDT synergy often facilitates total tumor eradication in fewer visits. Furthermore, the implementation of RCM or OCT during these combined procedures provides real-time, non-invasive monitoring to ensure that subclinical tumor nests are identified and treated, further bolstering long-term oncologic success [50]. Collectively, these combinatorial approaches appear to improve clinical outcomes while preserving surrounding tissue integrity.

## Discussion

This narrative review synthesizes the current clinical evidence regarding laser-based therapies for non-melanoma skin cancers, with an emphasis on practical parameters, patient/lesion selection, and follow-up considerations. Overall, lasers appear most applicable as tissue-sparing options for carefully selected low-risk lesions, particularly when cosmetic or functional preservation is a priority or when surgery is contraindicated. The clinical outcomes reported for laser therapies must be interpreted with significant caution. Most available studies focus on carefully selected, low-risk, superficial lesions, often with shorter follow-up durations compared to surgical trials. Crucially, the lack of histological margin control remains the most significant limitation, potentially leading to underestimated recurrence rates in deep-seated or aggressive tumor nests.

Among the modalities discussed, ablative CO₂ laser therapy has the broadest clinical experience for low-risk BCC. Reported outcomes are variable across series, reflecting heterogeneity in protocols, margins, and lesion characteristics; nevertheless, CO₂ may provide acceptable clearance and cosmetic outcomes in selected superficial or nodular lesions when applied with appropriate technique and surveillance.

The integration of RCM as an adjunct for pre-treatment mapping and post-treatment margin assessment may mitigate one of the main limitations of tissue-sparing laser approaches, the absence of standard histologic margin control, and may reduce the risk of persistence or early recurrence in anatomically complex sites.

Long-pulsed Nd: YAG lasers have also shown promising results for selected low-risk BCC. In the systematic review and meta-analysis by Sharon et al. [10], Nd: YAG was associated with a pooled recurrence rate of 3.1% during a mean follow-up of 7.9 years, although the evidence is still largely derived from observational studies and requires further prospective validation and protocol standardization [10].

In contrast, PDL treatment has been associated with higher recurrence and inconsistent efficacy across studies. The pooled recurrence estimate reported by Sharon et al. [9] (38%) and the variability by histologic subtype and parameters suggest that PDL should not be considered a reliable monotherapy for definitive treatment of BCC and should, at most, be reserved for highly selected scenarios or combined protocols.

For cSCC, the evidence is substantially less mature than for BCC. Most available data relate to cSCC in situ (Bowen’s disease) and to combination regimens (e.g., CO₂ plus PDT) or field-directed approaches. For invasive cSCC, due to the risk of metastasis and incomplete eradication in the absence of margin control, laser-based approaches for invasive disease are strongly discouraged outside of strictly controlled clinical trials. Therefore, the modest successes achieved must and can be attributed primarily to Bowen’s disease.

**Fig. 1:**
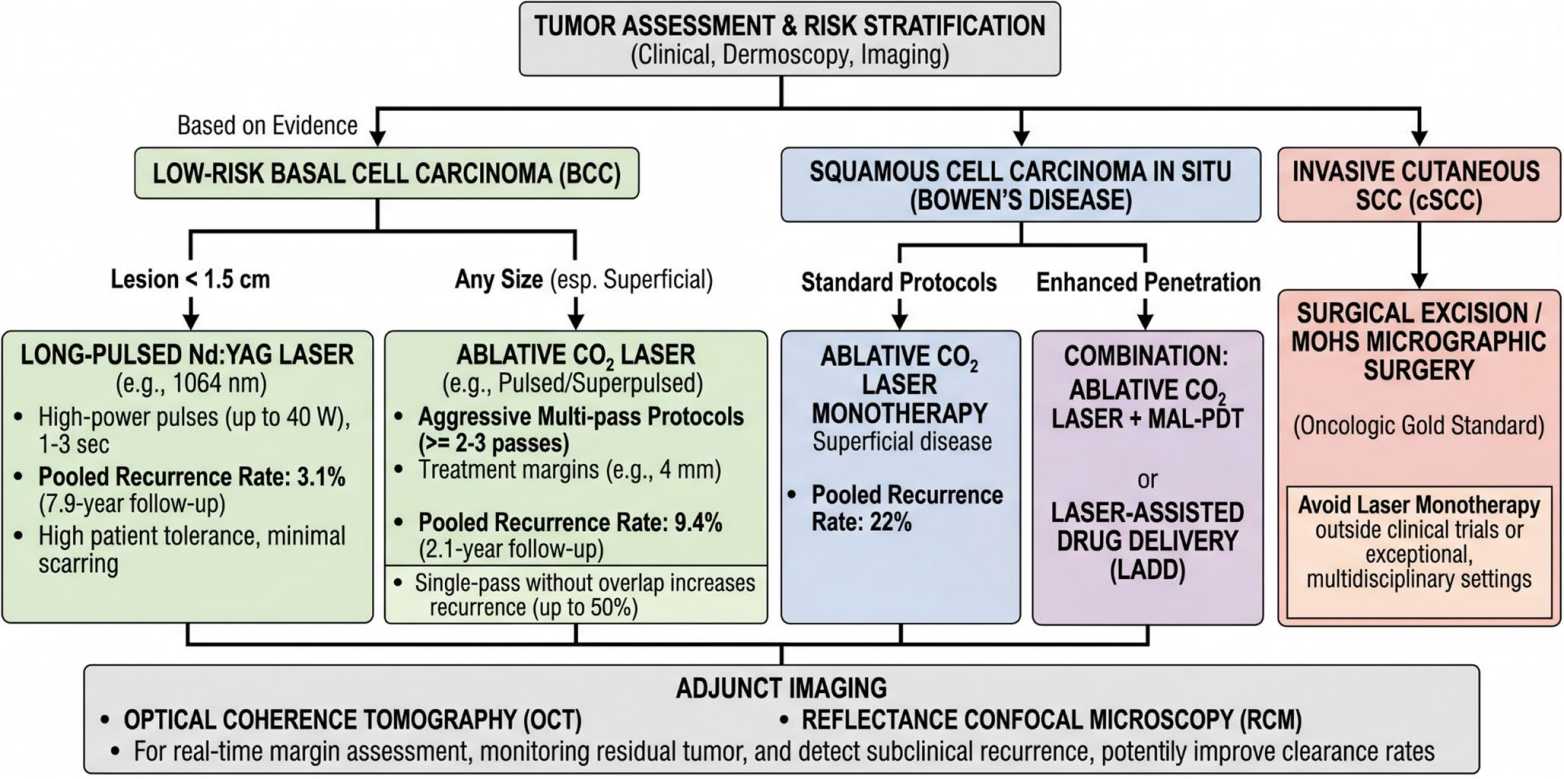
decision algorithm for laser-based management of non-melanoma skin cancer

Key limitations across the literature include small sample sizes, frequent reliance on single-arm cohorts, inconsistent reporting of laser settings and margins, heterogeneity in outcome definitions (clinical vs. histologic clearance), and variable follow-up. Long-term outcomes (beyond 5 years) remain insufficiently characterized for many protocols. Figure 1 offers a flowchart to guide clinicians choosing the right strategy.

Moreover, although cosmetic outcomes are frequently described as favorable, few studies use validated patient-reported outcome measures or standardized aesthetic scales, limiting cross-study comparability. Future studies should incorporate harmonized endpoints, standardized parameter reporting, and prospective follow-up to better define the role of lasers relative to established surgical and non-surgical therapies. Overall, laser therapy cannot replace surgery as the standard of care for high-risk, deeply infiltrative, or recurrent NMSC. Surgical excision and Mohs micrographic surgery remain the undisputed oncologic gold standard for NMSC, providing definitive histological margin assessment and the lowest recurrence rates. Laser therapies should be considered only as secondary options for patients who are not candidates for surgery or for specific low-risk superficial lesions. However, in selected low-risk lesions and in patients for whom surgery is undesirable or contraindicated, laser-based approaches, especially when integrated with adjunctive imaging and combination strategies, may offer clinically meaningful, tissue-sparing alternatives. As technology continues to advance, particularly in real-time imaging, device engineering, and translational photothermolysis, the role of lasers in dermatologic oncology is likely to expand. The priority for future research is to define standardized protocols and comparative effectiveness against surgery and established nonsurgical modalities across clearly defined risk strata.

## Conclusion and clinical recommendations

Laser therapy represents an evolving option for selected cases of non-melanoma skin cancer. For carefully selected low-risk BCC, ablative CO₂ protocols and long-pulsed Nd: YAG lasers may achieve meaningful clearance with favorable cosmetic outcomes, particularly in cosmetically or functionally sensitive areas, provided that protocol parameters are appropriate and follow-up is rigorous.

For cSCC in situ (Bowen’s disease), combination strategies such as CO₂ laser followed by PDT, as well as fractional laser-assisted topical therapies (LADD), may be considered in patients who are poor surgical candidates or who decline surgery. For invasive cSCC, the current evidence does not support definitive laser monotherapy outside of exceptional scenarios or clinical research. The reported efficacy rates of laser therapies must be interpreted with caution, as many studies involve small, retrospective cohorts of carefully selected superficial tumors .

In summary, lasers should be viewed as complementary tools within a risk-stratified, patient-centered treatment algorithm rather than as replacements for excision or Mohs surgery. Future prospective comparative trials with standardized parameter reporting and long-term surveillance are required to establish optimal indications, protocols, and monitoring strategies.

## Data Availability

No datasets were generated or analysed during the current study.
